# Supplementary material for: Traumatised Children’s Perspectives on Their Lived Experience: A Review
Source: Behav Sci (Basel). 2023 Feb 14;13(2):170. doi: 10.3390/bs13020170 (PMC9952495; doi:10.3390/bs13020170)
Supplement: Supplementary file 1 [file behavsci-13-00170-s001.zip › behavsci-2129351-supplementary.pdf]

Supplementary Table S1. Quality appraisal of studies reviewed.

	Questions of CASP checklist									
Authors (year of study)	1	2	3	4	5	6	7	8	9	10
Asgari and Naghavi (2020)	Y	Y	?	Y	Y	Y	Y	Y	Y	Y
Cherewick et al. (2015)	Y	Y	Y	Y	Y	Y	Y	Y	Y	Y
Chester and Joscelyne (2021)	Y	Y	Y	Y	Y	Y	Y	Y	Y	Y
Darcy et al. (2014)	Y	Y	Y	Y	Y	Y	Y	Y	Y	Y
Egberts et al. (2020)	Y	Y	?	Y	Y	Y	Y	Y	Y	Y
Figge et al. (2020)	Y	Y	Y	Y	Y	?	Y	Y	Y	Y
Foster (2017)	Y	Y	Y	Y	Y	Y	Y	Y	Y	Y
Foster and Hagedorn (2014)	Y	Y	Y	Y	Y	Y	Y	Y	Y	Y
Harazneh et al. (2021)	Y	Y	Y	Y	Y	Y	Y	Y	Y	Y
Jensen et al. (2013)	Y	Y	Y	Y	Y	Y	Y	Y	Y	Y
Jones et al. (2021)	Y	Y	?	Y	Y	?	Y	Y	Y	Y
Kieffer-Kristensen and Johansen (2013)	Y	Y	?	Y	Y	Y	Y	Y	Y	Y
Lee et al. (2018)	Y	Y	?	Y	Y	?	Y	Y	Y	Y
Lovato (2019)	Y	Y	Y	Y	Y	?	Y	Y	Y	Y
McGarry et al. (2014)	Y	Y	Y	Y	Y	Y	Y	Y	Y	Y
Parsons et al. (2021)	Y	Y	Y	Y	Y	?	Y	Y	Y	Y
Rohleder et al. (2017)	Y	Y	?	Y	Y	?	Y	Y	Y	Y
Salawali et al. (2020)	Y	Y	Y	Y	Y	Y	Y	Y	Y	Y
Tyerman et al. (2019)	Y	Y	Y	Y	Y	?	Y	Y	Y	Y

Note. CASP = Critical Appraisal Skills Programme. 1 = Was there a clear statement of the aims of the research? 2 = Is a qualitative methodology appropriate? 3 = Was the research design appropriate to address the aims of the research? 4 = Was the recruitment strategy appropriate to the aims of the research? 5 = Was the data collected in a way that addressed the research issue? 6 = Has the relationship between researcher and participants been adequately considered? 7 = Have ethical issues been taken into consideration? 8 = Was the data analysis sufficiently rigorous? 9 = Is there a clear statement of findings? 10 = How valuable is the research? Y = Yes. N = No. ? = Cannot tell.

Supplementary Table S2. Themes, subthemes, and examples of illustrative verbatim quotes.

Themes	Subthemes	Verbatim quotes
Daily life problems related to trauma	Daily routines	'I couldn't sleep or eat for weeks after my father got taken away from us.' (Lee et al, 2018)
	Relationship issues	'I was in hospital for ages....I didn't see like my sister, my dad, my brothers for quite a while. And my pets.' (Jones et al., 2021)
	Daily function	'Yeah, because normally you can do stuff on your own but when accidents happen like this, you're just left and everyone else is doing everything for you, which is frustrating.' (McGarry et al., 2014)
Negative responses to trauma	Physical aspect	"Days after the incident, I experienced bad conditions in my body. For example, I could not sleep well at night. I lost my appetite and felt severe weakness, heart palpitations, or tightness in my chest." (Asgari and Naghavi, 2020)
	Psychological aspect	'After the abuse I felt mad. After weeks I felt angry.' (Foster, 2017)
	Cognitive aspect	'At first I had really bad flashbacks, but now it has gotten less. But if I see someone now, say, on television and they're on fire, then I really do still get flashbacks.' (Egberts et al., 2020)
	Behavioural aspect	'I would either break the window, hurt my sister or do anything that made my parents upset, like really really upset.' (Parsons et al., 2021)
	Social aspect	'You know I got walls, I don't like telling people about things, cos I don't want to get hurt. I don't really like people knowing a lot about me.' (Chester and Joscelyne, 2021).
Perceived health needs	Emotional support	'Maybe I could have done it alone, but I was really glad my mother was with me. Just a familiar face with me. My mother also saw me in pain then, she also knows what I looked like then.' (Egberts et al., 2020)
	Social support	'They don't understand it, and I've always been very disappointed in them because I felt they were not there for me when I needed it.' (Kieffer-Kristensen and Johansen, 2013).
	More detailed information	'What's she going to be like cos I know she's not very, we don't know whether it's going to go downhill or if it's going to stay as it is, so it would be nice to know.' (Rohleder et al., 2017)
	Need for play	When we played games it helped me to not think of what had happened and I felt better' (Jensen et al.,

		2013)
Coping strategies related to trauma and stress	Cognitive coping strategies	'I thought about my friends, I tried to think about something else, that I was somewhere else and that I was playing soccer, or at least that I was doing something else than being there.' (Jensen et al., 2013)
	Behavioural coping strategies	'It feels really good talking about it, to kind of let all your inner thoughts out.' (Kieffer-Kristensen and Johansen, 2013)
Growth from traumatic experience	Meaning of life	'I was lazy. After that incident, I got guidance to be better. I used niqab (veil). Honestly, there are a lot of difficulties. Some people say don't be such a saint, and hypocrite. But I'm still patient because we live solely looking for the pleasure of God, not human. From all of this, I learned.' (Salawali et al., 2020)
	Close relationship	'My relationship with my family has become a lot better since the incident; I can say that we've become much more intimate than before. We spend more time together. We love each other much more than before.' (Asgari and Naghavi, 2020)
	New life goals	'Yeah, because it sort of changed everything because now I'm thinking that I should maybe be a burn doctor. I want to help other people.' (McGarry et al., 2014)
	Personal strengths	'My power to endure problems has increased. When something bad happens, you will have higher tolerance towards other life difficulties.' (Asgari and Naghavi, 2020)
	Religious beliefs	"I feel my faith is stronger now. Sometimes when I have a problem and I don't know what to do, I feel God's presence with all my heart – as if someone is helping me overcome my problems. My relationship with God is much better than before." (Asgari and Naghavi, 2020)
